# Identification of a Unique Morphological Pattern for the Diagnosis of Fungal Rhinosinusitis

**DOI:** 10.7759/cureus.41915

**Published:** 2023-07-15

**Authors:** Maheen Maruf, Asif Loya, Sajid Mushtaq, Usman Hassan, Mudassar Hussain, Maryam Hameed

**Affiliations:** 1 Histopathology, Shaukat Khanum Memorial Cancer Hospital and Research Centre, Lahore, PAK

**Keywords:** granulomatous fungal rhinosinusitis, gms, crowded giant cell pattern, aspergillus, granulomatous, fungal rhinosinusitis

## Abstract

Fungal rhinosinusitis (FRS) is a relatively common, but often misdiagnosed disease of paranasal sinuses. The FRS is classified into invasive and non-invasive forms. The non-invasive form includes fungal ball and allergic FRS, and invasive form includes acute invasive FRS, chronic invasive FRS, and granulomatous FRS. Invasive fungal infections are associated with high morbidity and mortality, hence requiring urgent medical and surgical intervention. The histomorphology can help identify certain fungal organisms that cannot be cultured or are rarely visible in exudates. The morphologic diagnosis of tissue invasive and non-invasive fungal infection is essential for appropriate treatment.

We analyzed cases of rhinosinusitis from 2017 to 2019 in Pathology Department at a tertiary care cancer hospital, Lahore, Pakistan. All clinical information was retrieved from patient records. Paraffin-embedded tissue blocks were stained with hematoxylin and eosin (H&E), special Grocott methenamine silver stain (GMS), and periodic acid Schiff stain (PAS) according to standard protocol. They were reviewed by two pathologists blinded by fungus status.

A total of 169 cases of rhinosinusitis were reviewed. FRS comprised 146 (86.4%) of them. The mean age of patients with FRS was 32.8±14 years. The male:female ratio was 1.4:1. Maxillary sinus was the main site of involvement in 39 (27%) FRS cases. Aspergillus was identified in 117 (80.1%) cases of FRS. The culture reports were available in 44/146 (30.14%) FRS cases. They were negative in 22/44 (50.0%), and Aspergillus species were isolated in 18/44 (40.9%) cases of FRS.

There were 84 (57.5%) cases of non-invasive FRS and 59 (40.4%) cases of invasive FRS. Among invasive FRS, there were 56 (38.4%) chronic granulomatous FRS cases including mixed patterns. Majority cases, 54 (96.4%), of chronic granulomatous FRS showed a unique crowded giant cell pattern comprising of foreign body and Langhans type giant cells. These giant cells were arranged closely forming irregular non-caseating granulomas surrounded by lymphocytes and fibrosis. Interestingly, the giant cells were scattered haphazardly without forming a granuloma as well. Fungal organisms were identified in all 56 cases of chronic granulomatous FRS. Histologically, predominant organism was Aspergillus in 48 (85.7%) on GMS and PAS stain.

Our study observed a unique crowded giant cell pattern, which is a hallmark of invasive fungal infection. If pathologists are familiar with this unique pattern, they can make a quick and accurate diagnosis on histology. The physician can start antifungal treatment timely for better prognosis.

## Introduction

Rhinosinusitis commonly affects 20% people. Acute rhinosinusitis is self-limited and caused by upper respiratory tract viral or bacterial infections [1]. Chronic rhinosinusitis is a heterogenous disease. It has many phenotypes including fungal rhinosinusitis (FRS), infectious rhinosinusitis, aspirin exacerbated rhinosinusitis, pediatric rhinosinusitis, and rhinosinusitis associated with systemic disease [2]. Chronic rhinosinusitis has a protracted course, caused by fungal organisms in majority cases [1].

FRS is classified into invasive and non-invasive forms. Non-invasive FRS includes allergic and fungal ball, whereas invasive FRS includes acute, chronic, and granulomatous [1,3]. This is based on type of inflammatory tissue reaction and duration of disease. Fungal ball shows mild mucosal inflammation, whereas allergic FRS is characterized by eosinophilic mucin and fungal elements. Acute invasive FRS is usually seen in immunocompromised hosts, has a rapid clinical course, and can invade orbit and brain. There is abundance of chronic inflammatory cells in chronic invasive FRS and granulomatous reaction in granulomatous FRS due to tissue invasive fungal organisms [4].

Invasive fungal infections of the head and neck are more common than reported [1]. Head and neck (sinonasal cavity) is a source of entry and colonization of fungal organisms [5]. They may present with complains of nasal obstruction, rhinosinusitis, or recurrent nasal polyps [1]. Their diverse presentation makes the diagnosis difficult [4]. A high index of suspicion will result in early diagnosis and management. Invasive fungus will require urgent surgical debridement and appropriate antifungal therapy [6].

Fungal cultures are considered a gold standard for the identification of species and supplementing histology diagnosis. But culture studies are time-consuming and may not always be available or grow the fungal organism [7,8]. Some fungal forms may grow rapidly invading vital structures (orbit, nasal cavity, and vessel wall) especially in the head and neck region. Their rapid diagnosis is essential for initiating timely treatment and reducing morbidity and mortality [3].

There are other techniques for the diagnosis of fungal infection, such as in situ hybridization (ISH) to detect ribosomal ribonucleic acid (rRNA) of fungi, immunoassay to detect fungal antigens, genomic amplification, and serology to detect immunoglobulins against fungi [9]. These are expensive, not readily available especially in developing countries, and have decreased utility in necrotic tissue [3,4,8].

The clinical features can give a clue of subcategories of FRS; however, morphological diagnosis is more accurate [1]. Histopathologic diagnosis has higher sensitivity (76.67%) than culture studies (50%) [6]. Ravindra and Viswanatha showed fungal organisms in 12/60 (20%) rhinosinusitis cases, while culture studies were positive in only 4/12 (33%) FRS cases [10]. Histopathology is a rapid, accurate, and cost-effective method of diagnosing fungal infection. The histomorphologic pattern can help identify certain fungal organisms that cannot be cultured or are rarely visible in exudates such as rhinosporidiosis and pneumocystis [8]. The morphologic diagnosis of tissue invasive and non-invasive fungal infection is essential for appropriate treatment [1].

Previously, the most landmark research on FRS was conducted in Europe and the United States of America [6]. Hora classified sinus mycosis in invasive and non-invasive forms in 1965. Mc Gill reported four cases of sinonasal fulminant aspergillosis in 1980 and De Shazo et al. published classification of invasive and non-invasive FRS in 1997 [4]. There is paucity of data regarding type and histologic pattern of head and neck fungal infection in our country. Therefore, our study aims to identify the type and morphological pattern of head and neck fungal infections in the Pakistani population.

## Materials and methods

The study included a total of 169 retrospective cases of rhinosinusitis, diagnosed between January 2017 and December 2019 in the Pathology Department at Shaukat Khanum Memorial Cancer Hospital and Research Center, Lahore, Pakistan. Cases of both genders and all ages of rhinosinusitis were included. Insufficient tissue for diagnosis, other body sites of fungal infections, and malignancy were excluded. All clinical information such as age, sex, and sites were retrieved from patient records including culture reports. This research project was approved by Institutional Review Board (IRB number EX-24-03-20-03, dated April 24, 2020) at Shaukat Khanum Memorial Cancer Hospital and Research Center. The patient data confidentiality was in line with the Declaration of Helsinki [11].

All paraffin-embedded tissue blocks were stained with hematoxylin and eosin (H&E) and special stains Grocott methenamine silver stain (GMS) and periodic acid Schiff stain (PAS) according to standard protocol [12].

The tissue sections were stained using an H&E staining kit (Leica ST 5020). Paraffin-embedded sections were dewaxed with xylene, hydrated with decreasing gradient ethanol, and stained with H&E stain. Then the sections were rehydrated in increasing gradient ethanol, cleared with xylene, and mounted on glass slide.

The sections were stained with GMS using GMS staining kit (Agilent Dako Artisan Link Pro, Agilent Technologies, Santa Clara, CA). Paraffin-embedded sections were dewaxed with xylene and gradient ethanol, oxidized in 8% chromic acid solution for 20 minutes, and then treated with 0.5% sodium metabisulfite solution; the sections were immersed in methenamine silver nitrate solution preheated at 60 ° C and placed in an electrothermostatic blast oven at 60 ° C for 40 minutes. The sections were washed, treated with 5% sodium thiosulfate solution, and counter-stained with light green solution. The sections were rehydrated in gradient ethanol, cleared with xylene, and mounted on glass slide. The fungal organism showed brownish black staining in a light green background.

The sections were stained with PAS using PAS staining kit (Ventana Benchmark Special Stains, Roche, Basel, Switzerland). Paraffin-embedded sections were dewaxed with xylene and gradient ethanol and then oxidized with 1% periodic acid solution for 10 minutes. Then treated with Schiff reagent in dark for 20 minutes. The sections were washed with water for 5 minutes and counter-stained with hematoxylin. The sections were rehydrated in gradient ethanol, cleared with xylene, and mounted on glass slide. The fungal organism showed magenta color.

All the tissue sections were reviewed by two pathologists blinded by status of fungal organism. This enabled to detect and classify rhinosinusitis on the presence of allergic mucin, fungal organism, and tissue response [8].

The data were analyzed in Microsoft Excel. Mean and standard deviation were calculated for quantitative variables such as age. Frequency and percentage were calculated for categorical variables such as gender and frequency of FRS.

## Results

A total of 169 cases of rhinosinusitis were reviewed. FRS comprised 146 (86.4%) of them. The remaining 23 (13.6%) were non-FRS cases. The mean age of patients with FRS was 32.8±14 years. There was more male, 84 (57.53%), preponderance than female, 62 (42.47%). The male:female ratio was 1.4:1.

**Figure 1:**
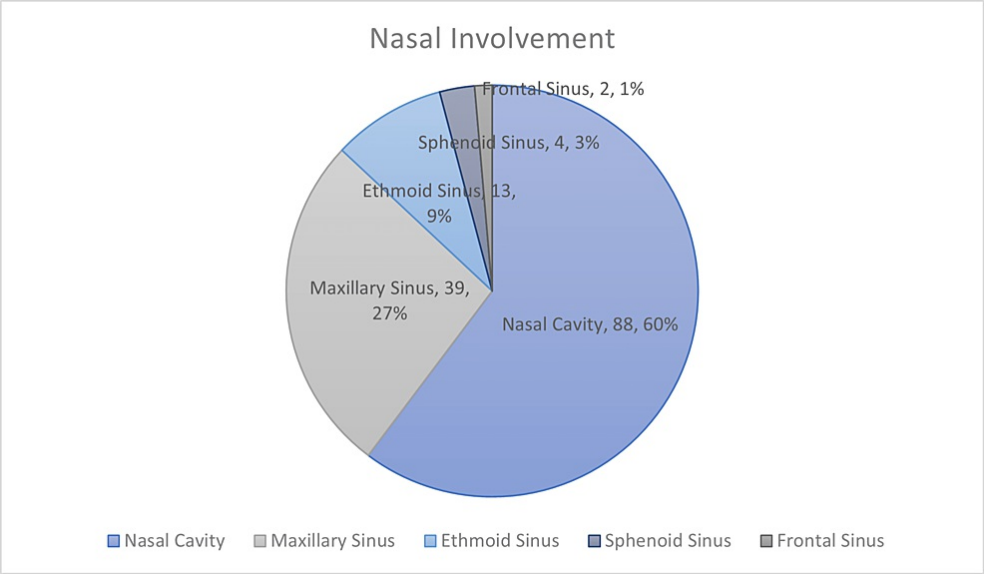
Sinonasal involvement in fungal rhinosinusitis

The isolated nasal cavity was involved in 88 (60%) FRS cases. However, maxillary sinus showed predominant involvement, with 39 (27%) cases, as opposed to frontal sinus, with two (1%) cases (Figure 1).

**Figure 2:**
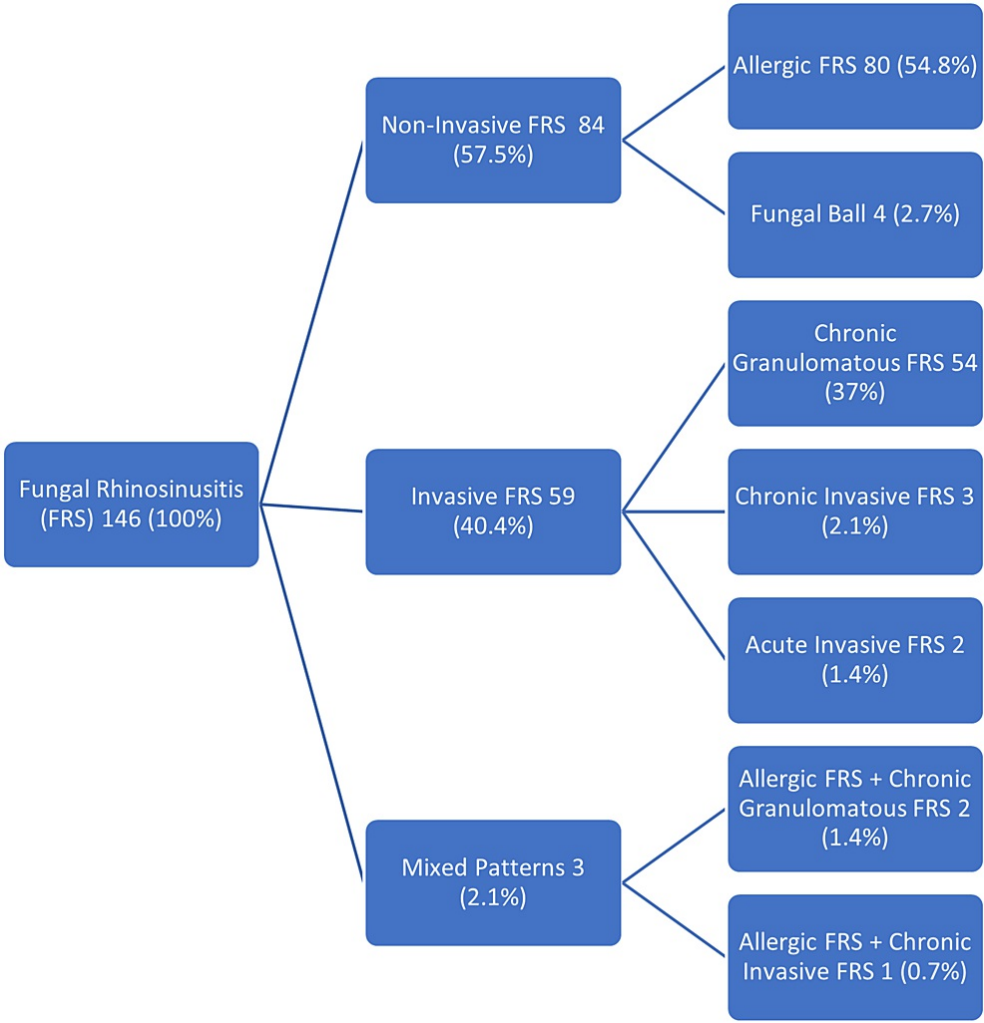
Observed types of fungal rhinosinusitis

We broadly classified FRS cases on histological examination into non-invasive FRS, invasive FRS, and mixed patterns (Figure 2) [1,10].

**Figure 3:**
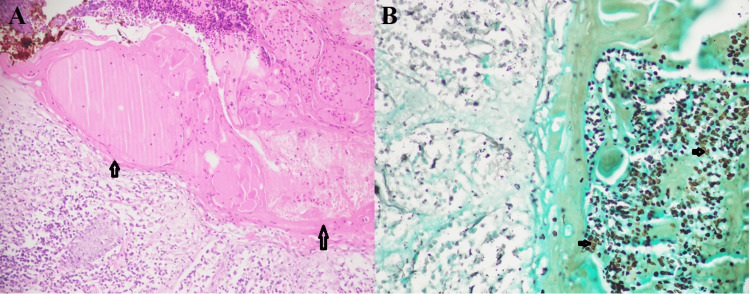
Allergic fungal rhinosinusitis A. Areas of allergic mucin (arrows) with sloughed epithelial cells, eosinophils, and inflammatory debris (H&E 20X). B. Round small black candida spores (arrows) in allergic mucin (GMS 40X).

Non-invasive FRS comprised predominantly allergic FRS, with 80 (54.8%) cases. The allergic FRS was characterized by eosinophilic mucin admixed with slough epithelial cells, eosinophils, and inflammatory cells in a laminar pattern (Figures 3A, 3B).

**Figure 4:**
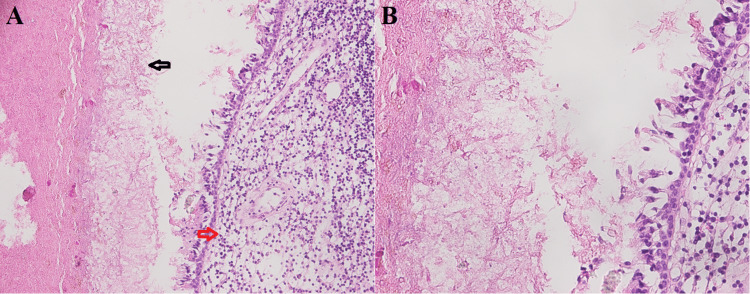
Fungal ball A. Tightly packed fungal hyphae (black arrow) in necrotic exudate with mild mucosal inflammation (red arrow, H&E 20X). B. Septate fungal hyphae (*Aspergillus*) visible on H&E (40X).

Fungal ball showed entangled mass of fungal organisms embedded in fibrinous necrotic exudate in four (2.7%) cases. Minimal or no mucosal inflammatory reaction was also noted (Figures 4A, 4B).

Our study showed that invasive FRS mostly comprised of chronic granulomatous invasive FRS, 56 (38.4%), including mixed pattern (Figure 2). We identified a unique submucosal crowded giant cell pattern in 54/56 (96.4%) cases of chronic granulomatous invasive FRS including mixed pattern. All cases revealed fungal organisms either on H&E or special stains. Morphologically, the crowded giant cell pattern showed two types of giant cells: Langhans and foreign body type giant cells. The Langhans type giant cells have 10-15 nuclei arranged in a horseshoe shape at one pole. The foreign body giant cells have a haphazard nuclear arrangement. The size of these giant cells was variable (40-200 µm) [13]. There were at least three to four giant cells crowded together surrounded by epithelioid histiocytes, lymphocytes, and fibrosis, forming small irregular granulomas (Figure 6A, 6E). These granulomas showed no necrosis. An interesting feature was that these giant cells can be scattered irregularly without forming a granuloma as well (Figure 6D). Histologically, septate fungal hyphae were identified as *Aspergillus* in 48 (85.7%) cases, broad ribbon-like aseptate hyphae as *Mucor* in three (5.4%) cases, slender pseudo hyphae and spores as candida in one (1.8%) case, and both *Aspergillus* and *Mucor* in two (3.6%) cases on GMS and PAS stain within the giant cells and scattered in the granuloma.

**Figure 5:**
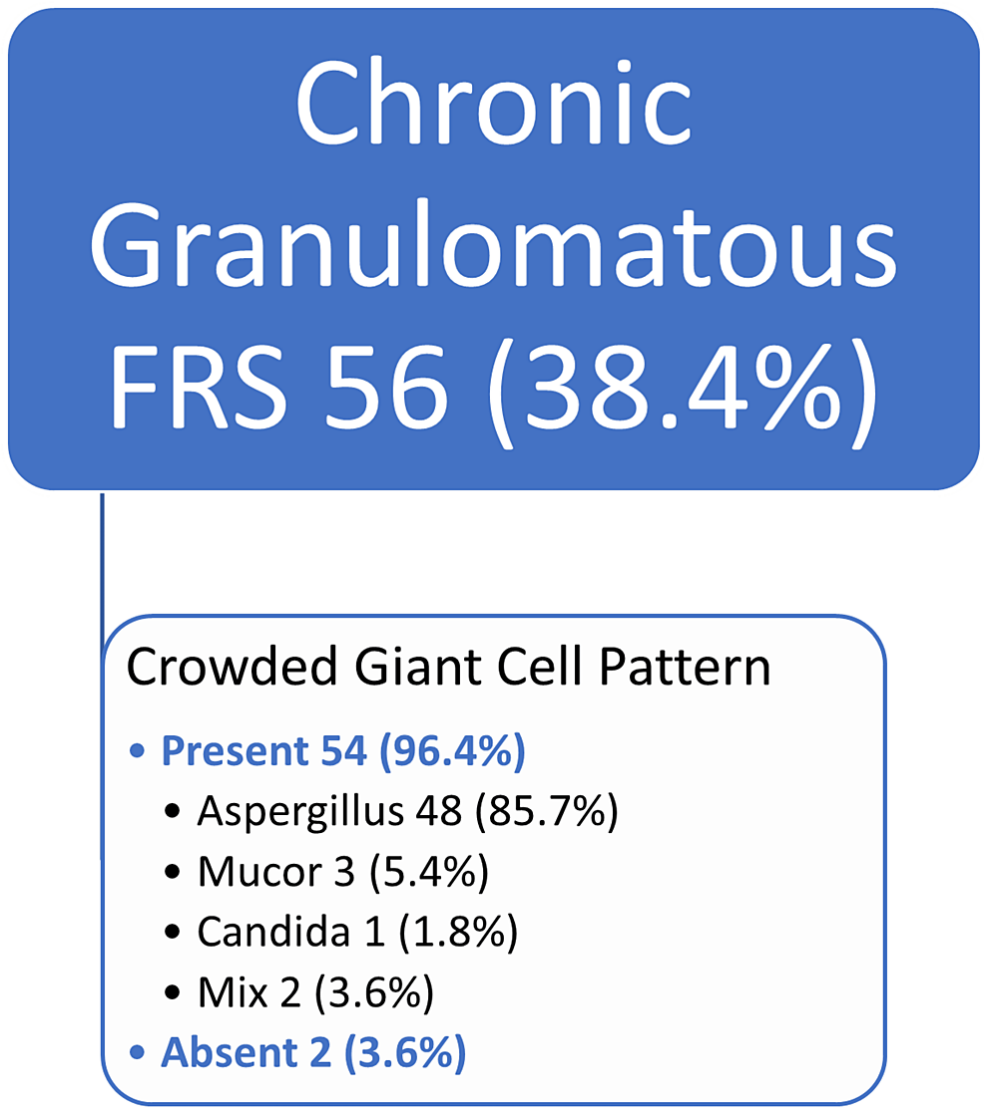
Unique crowded giant cell pattern in chronic granulomatous FRS FRS, fungal rhinosinusitis

**Figure 6:**
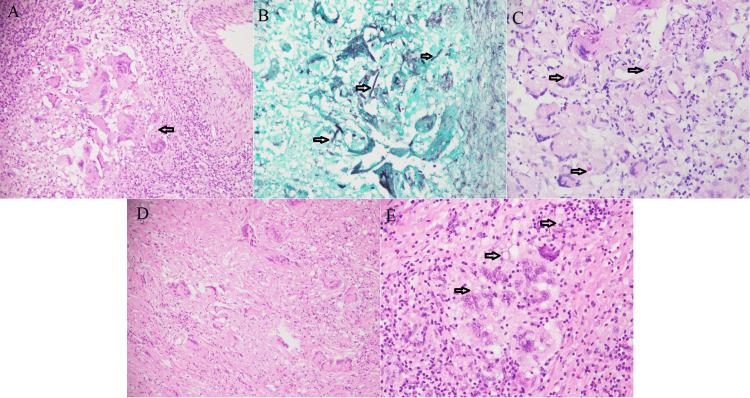
Unique crowded giant cell pattern A. Small irregular submucosal granuloma (arrow) showing Langhans and foreign body type giant cells crowded together (H&E 20X). B. Septate fungal hyphae (*Aspergillus*, arrows) in giant cells and scattered in granuloma (GMS 40X). C. *Aspergillus* (arrows) in giant cells (PAS 40X). D. Giant cells scattered haphazardly, not forming a granuloma (H&E 20X). E. Fungal spores (arrows) are visible within the giant cell (H&E 40X).

Only 2/56 (3.6%) showed characteristic smaller Langhans giant cells; however, crowded giant cell granulomatous pattern could not be convincingly appreciated. This might be due to scanty viable tissue in biopsy sample. But their GMS stain showed fungal organism (Figures 5, 6A-6E).

**Figure 7:**
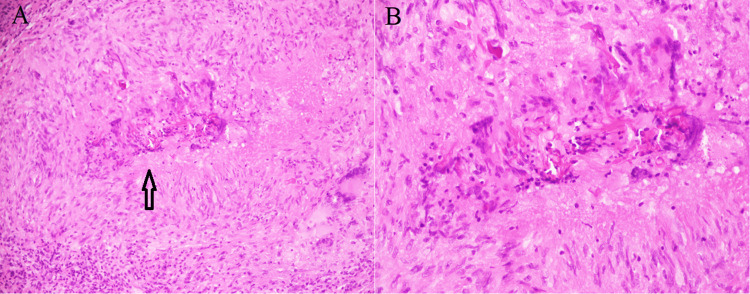
Chronic invasive fungal rhinosinusitis A. Splendor Hoeppli reaction (asteroid bodies, arrow), acute inflammation, necrosis, and scattered giant cells (H&E 20X). B. Splendor Hoeppli reaction: intensely eosinophilic material radiating star-like around ribbon-like aseptate fungal hyphae (*Mucor*) (H&E 40X).

Chronic invasive FRS showed tissue invasive fungal hyphae surrounded by an eosinophilic Splendor-Hoeppli reaction, acute inflammation, necrosis, and scattered sparse giant cells in three (2.1%) cases (Figures 7A, 7B).

**Figure 8:**
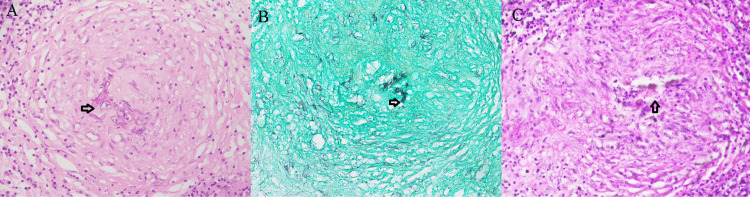
Acute invasive fungal rhinosinusitis A. Vascular thrombosis and fibrosis with broad aseptate fungal hyphae (arrow) in the vessel wall and sparse inflammation (H&E 40X). B. Broad ribbon-like fungal hyphae (*Mucor*, arrow) (GMS 40X). C. Focal coagulative necrosis with magenta-colored *Mucor* hyphae (arrow) on PAS (40X).

The acute invasive FRS was characterized by extensive coagulative necrosis, scant inflammatory reaction and angio-invasive fungal hyphae in two (1.4%) cases (Figures 8A-8C).

**Figure 9:**
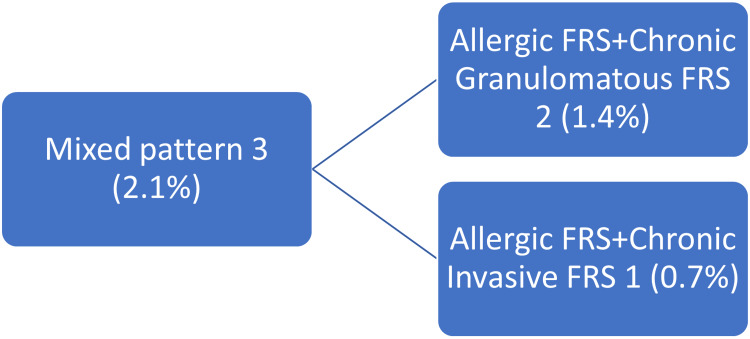
Mixed pattern of fungal rhinosinusitis

The mixed pattern comprising features of both invasive and non-invasive FRS consisted of three (2.1%) cases (Figure 9).

**Figure 10:**
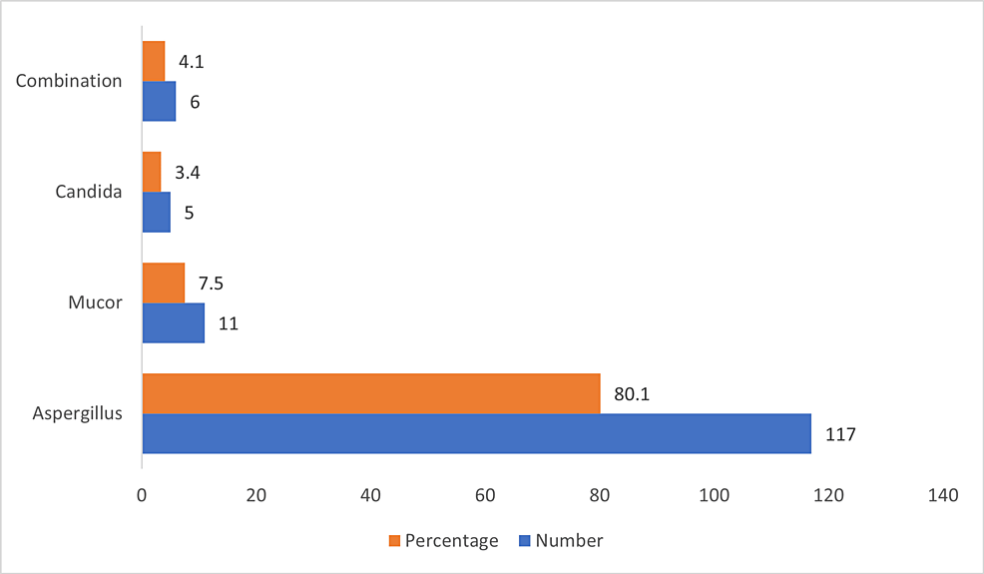
Morphologically identified fungal organism in FRS cases FRS, fungal rhinosinusitis

Morphologically, *Aspergillus *was commonest overall in FRS cases, with 117 (80.1%) cases. The combination of *Aspergillus *and *Mucor* was seen in six (4.1%) cases (Figure 10).

The culture report was available in 44 (30.14%) cases of FRS. It was negative in 22 (50.0%) cases, whereas isolated *Aspergillus* species was found in 18 (40.9%) cases, *Fusarium* species in one (2.3%) case, and *Scopulariopsis brumptii* in one (2.3%) case among them.

## Discussion

Fungal infections range from superficial corneal lesions to systemic involvement. These infections are commonly seen in immunocompromised individuals such as transplant recipients, cancer and HIV (human immunodeficiency virus) patients, premature babies, and elderly individuals. However, they are capable of infecting healthy individuals as well [8].

FRS previously considered rare is being increasingly reported now [1]. Our study showed 146 (86.4%) FRS cases. Previously, studies have shown an incidence of 284 (42.7%) [1] and 37 (48.7%) [14] cases of FRS, which is lower than our study. The increased prevalence of FRS in our population can be due to sedentary lifestyle causing metabolic syndromes (diabetes, obesity, hypertension), low socioeconomic status, poor hygiene, and living conditions [6].

In our study, the mean age of patients with FRS was 32.8±14 years. This is in concordance with the literature showing majority cases of FRS in the third and fourth decades of life [6,10,15]. Our study showed males, 84 (57.53%), to be predominantly affected by FRS. This is similar to studies with 22 (75.8%) male gender involvement [8] and 19 (63.3%) [6] male preponderance.

In our study, primarily the nasal cavity was involved, with 88 (60%) cases, followed by maxillary sinus, with 39 (27%) cases. This contrasts with a previous report showing predominant maxillary sinus involvement in 10 (34.6%) cases, followed by nasal cavity in nine (31.1%) cases [8]. Another study showed a higher incidence of maxillary sinus involvement, with 14 (46.6%) cases [6].

Histologic examination is a fast and cheap method for the diagnosis of fungal infection. It can confirm the presence of fungus and tissue invasion. Early diagnosis is important in immunocompromised patients [9]. The infection can spread to the surrounding tissue (orbit, cavernous sinus, and meninges). This can cause visual impairment, intracranial extension, and even death [16].

There were 84 (57.5%) cases of non-invasive FRS, which comprised of 80 (54.8%) cases of allergic FRS and four (2.7%) cases of fungal ball. Detection of fungal hyphae in allergic mucin is important for the diagnosis of allergic FRS [1]. Allergic mucin is inflammatory exudate formed by mucus with eosinophil clumps. Fungal ball presents as unilateral nasal obstruction in middle-aged women as maxillary sinusitis [2]. This can easily be recognized on routine H&E stain [1].

Fungal hyphae penetrate the mucosa of nose and paranasal sinuses in invasive fungal sinusitis [17]. Studies have shown that the elderly, those with intracranial extension, and patients on conservative management have a poor prognosis [2].

Our study showed 56 (38.4%) cases of chronic granulomatous FRS including mix patterns. Similar results are reported by Alotaibi et al., with six (35.3%) cases [17]. Another study showed 24 (29.6%) cases of chronic granulomatous FRS [15]. Chronic granulomatous FRS usually occurs in healthy individuals. Its incidence has increased in Pakistan, India, Sudan, Saudi Arabia, and some areas of the United States of America. It presents with nasal cavity mass lesions simulating malignancy [1].

We observed a unique submucosal crowded giant cell pattern in most (54 [96.4%]) of the chronic granulomatous FRS cases in our study. There were at least three to four Langhans and foreign body giant cells crowded together surrounded by lymphocytes and fibrosis forming small irregular granulomas with no necrosis. Interestingly, these giant cells can be scattered irregularly without forming a granuloma as well. GMS stain was positive for fungal organisms in all 56 (100%) cases of chronic granulomatous FRS.

The literature has shown chronic granulomatous FRS characterized by submucosal non- caseating granulomatous inflammation with foreign body and Langhan type giant cells, fibrosis, and fungal hyphae [3,4,18]. However, this crowded giant cell pattern is not described in the literature. The differential diagnosis of giant cell lesion in the head and neck region also include central or peripheral giant cell granuloma [14,17]. The recognition of this pattern can only be done by histologic evaluation [7]. If the pathologists are familiar with this unique pattern, they can prompt the physician to start antifungal treatment quickly for better prognosis, reducing morbidity and mortality. Special stains can be used for fungal specie identification especially at low power [7]. GMS is very sensitive stain, and it is recommended not to give a negative diagnosis of fungal etiology without performing GMS [4].

Septate fungal hyphae (*Aspergillus*) were commonly identified in 48 (85.7%) cases similar to prior studies [3]. This has invasive potential especially through thin lamina papyracea in the nasal cavity into the brain. It is important to have prompt diagnosis of fungal infection for initiation of treatment and better prognosis [9].

The crowded giant cell pattern could not be convincingly appreciated in two (3.6%) cases in our study. This might be due to scanty tissue, sparse fungal elements, biopsy taken from non lesional area, or interobserver variability. However, it still prompted us to perform GMS stain, which detected fungal hyphae. Fungal cultures are not helpful in the diagnosis of granulomatous FRS, cannot establish tissue invasion, and cannot differentiate between colonization and contamination [4,19].

In our study, the mixed pattern featuring both invasive and non-invasive FRS consisted of three (2.1%) cases. Among them, two (1.4%) cases were a combination of allergic FRS and chronic granulomatous FRS, and one (0.7%) cases was a combination of allergic FRS and chronic invasive FRS. Literature concurs this finding, hence raising the possibility that non-invasive FRS can progress to invasive form [1].

The most common fungal organism in FRS was *Aspergillus*, 117 (80.1%), followed by *Mucor*, 11 (7.5%), in our study. Morphologically *Aspergillus* has thin septate branching hyphae at 45°, contrary to broad aseptate irregular branching at 90 ͦ *Mucor *hyphae [8]. Diagnosis of etiologic agent is essential for the initiation of appropriate antifungal treatment. These results are in concordance with Ravindra and Viswanatha’s study showing 11 (91%) cases of *Aspergillus* and one (8%) case of *Mucor* [10]. However, this contrasts with a study in which *Mucor* was common etiologic agent in 14 (48.3%) cases [8]. *Aspergillus* flavus is the major etiologic agent in allergic FRS, fungal ball, and chronic granulomatous FRS [1].

Culture studies are regarded as gold standard technique for the detection of fungal organisms. However, culture studies take time, and some fungi cannot be cultured. Differentiation between colonization and contamination can be problematic. Other methods do not detect all kinds of fungi and are not available at majority medical centers in low socioeconomic countries. Histologic method can detect majority fungal organisms, even those that cannot grow on culture media. It can be fast and economical. The tissue invasion and inflammatory response can be evaluated. Therefore, histologic examination is an important diagnostic tool in FRS [8].

The fungal culture studies were negative in 22 (50.0%) cases. The discrepancy between negative cultures and positive GMS stain for fungal hyphae can be explained by entrapped fungal hyphae in mucin. This prevents contact with culture medium. Other reason might be inappropriate tissue sampling [1]. Moreover, the importance of culture studies is decreased by false positivity seen in cases of fungal ball [9].

Our study showed microbiological cultures isolated *Aspergillus *species in 18 (40.9%) cases in majority; however, this contrasts with candida isolated in eight (26.6%) cases in another study [6]. This is possible due to their relatively smaller sample size as compared to ours.

FRS prevalence has increased in recent years [10]. This increase is owed to rise in immunocompromised individuals due to diabetes mellitus, acquired immunodeficiency syndrome, organ transplant, and cancer in our part of the world [5]. Early diagnosis is important for timely management, but clinical examination is seldom definitive [4]. An accurate diagnosis is required to omit excessive use of antibiotics and unnecessary surgery [10]. Histopathologic diagnosis is a benchmark for the diagnosis of FRS. Culture studies are useful but require special conditions for obtaining positive results [9].

There are a few limitations of our study as well. Firstly, cases of rhinosinusitis were selected, which included FRS at a single center. Multicenter studies will further increase the sample size as well as geographical distribution. Secondly, we only studied FRS and observed the unique crowded giant cell pattern pathognomonic for invasive granulomatous FRS. Prevalence of fungal infections and the unique crowded giant cell pattern at other sites of body need further investigation.

## Conclusions

Fungal infections can affect both immunocompromised and healthy individuals. They can particularly be harmful for the former and spread to vital organs rapidly. FRS diagnosis depends on identification and distribution of fungal hyphae. Microbiologic culture studies can be helpful, but they are not always positive. The histologic examination provides rapid identification of fungal organism. This can decrease the morbidity and mortality of infected patients. Our study observed a unique crowded giant cell pattern, which supports invasive fungal etiology in rhinosinusitis. It can be diagnosed on histopathology only. If pathologists are familiar with this crowded giant cell pattern, they can reach a quick and accurate diagnosis. The physician can start antifungal treatment timely for better prognosis. Other modalities for diagnosis can be omitted saving resources especially in low socioeconomic countries.
